# Plant-Based Solutions for Non-Productive Sites Useful in the Management of Dry Land

**DOI:** 10.3390/plants12030537

**Published:** 2023-01-24

**Authors:** Ewa Hanus-Fajerska, Katarzyna Kępka, Cezary Kruszyna, Iwona Kamińska

**Affiliations:** 1 Department of Botany, Physiology and Plant Protection, Faculty of Biotechnology and Horticulture, University of Agriculture in Krakow, Al. 29 Listopada 54, 31-425 Kraków, Poland; 2 PhD Candidates School, University of Agriculture in Krakow, Al. Mickiewicza 21, 31-120 Kraków, Poland

**Keywords:** regenerative agriculture, populated areas, environmentally friendly techniques, plant choice

## Abstract

The article presents an overview of research conducted in recent years, i.e., from 2004 until now. The study has been prompted by the threat of drought over large land areas which, as a result of current climate change, may lead to desertification in dry and hot regions of the world. For the same reason, large areas of farmland are affected by drought stress. At the same time, rising air temperatures result in a significant intensification of evaporation and a gradual increase in soil salinity. This applies in particular to acres of farmland, forested areas, and green areas of cities, as well as degraded land or brownfields. As the crop stability is threatened, the food base of the world’s population is at risk and, additionally, in areas of industrial districts, people’s health is in decline. Due to these multistress conditions for plant growth, we propose a review of the current literature which addresses the possibility of counteracting these unfavorable phenomena through the appropriate selection of plant species and, when only applicable, also through specific agroecological treatments. A selection of herbaceous and woody plants useful for cultivation on saline marginal lands was proposed.

## 1. Introduction

The effects of climate change, especially the long-term increase in air [1] and water [2] temperatures, result in a significant frequency and simultaneous high intensity of extreme weather events occurring in recent decades. It was found that, over a large area of the globe, there was a significant extension of rainless periods [3,4]. The currently observed changes in the water balance, resulting from a drastic modification of the distribution of precipitation, are a direct cause of drought threats and, at the same time, flood episodes, the so-called multi-hazard events [5,6,7]. Those hydroclimatic hazards affect biomes on all continents. 

Responses of vegetation representatives to those stress factors are very complex [8,9,10,11], and may be aggravated by long-term episodes of such events. The drought stress has been proven to be particularly severe for plants growing in marginal areas [12,13]. In addition, irregular and unpredictable precipitation combined with unfavorable properties of soil, high insolation, and high air and soil temperatures, significantly deepen the stress experienced by plant species [14,15].

Due to the importance of the threat of long-term drought in large areas of the mainland, in this paper we address the synthetized literature concerning cultivation in marginal areas. We discuss and propose some ways of protecting soils in drought-prone areas. Finally, we take up the problem of the appropriate selection of plant material for over-dried saline soils, which are particularly difficult to manage. The article presents an overview of research conducted in recent years, i.e., from 2004 until now.

## 2. Are We in Real Danger of Desertification of the Earth’s Surface?

### 2.1. The Importance of Anti-Crisis Management

Forecast scenarios indicate that a steady increase in average annual air temperature of about 1.5–2 °C is still expected in the coming decades. The number of days with temperatures below the annual average is expected to drop by half for a given area, while the number of days with maximum temperatures will double [1,16]. In vast areas on a global scale, the frequency of prolonged droughts will be increasing and, at the same time, the frequency of hurricanes and floods will increase, mainly in coastal areas [17,18]. In each case described, these are not suitable conditions for undisturbed plant growth and development. Therefore, the food base of the human population is being seriously threatened [19,20,21]. For this reason, research work on the response of plants to multi-stress conditions is constantly being undertaken [22], and attempts are being made to obtain lines with an increased degree of tolerance to various types of abiotic stresses [23,24]. 

The most important activity, which is necessary to be able to effectively counteract these extreme threats, is the continuous improvement of anti-crisis management [25,26] in order to provide a greater degree of environmental, economic and food security for the human population [27]. At the local level, special attention should be paid to protecting water resources and storing rainwater during the rainy season, whereas proper land use and environmental planning is an extremely important aspect of spatial development [28,29]. A good example would be the European economic development strategy, which envisages some increases in investment in the agricultural, forestry and processing sectors [30,31]. This is due to a predicted decrease in rainfall, which is likely to affect southern and central areas of Europe. Furthermore, in this vast area the drought will be prolonged and more severe due to rising temperatures.

### 2.2. Different Levels of Drought

**Figure 1:**
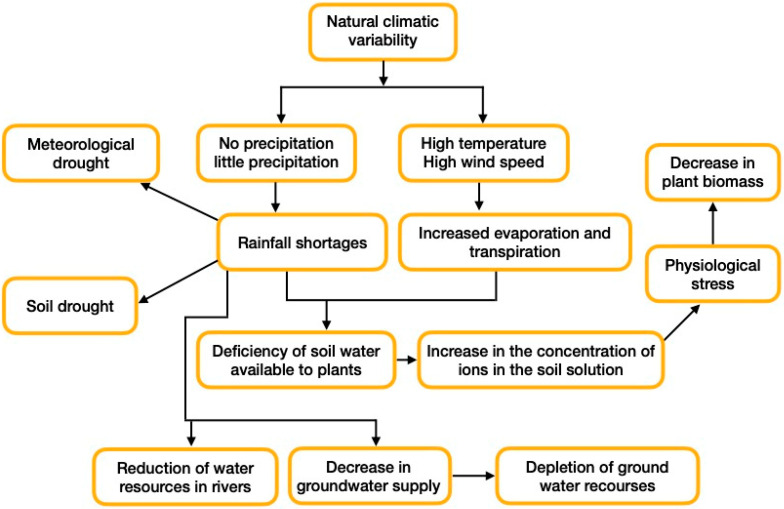
A brief graphic overview depicting the different phases of the hydrological cycle leading to drought, which generates a significant stress response in plants.

Drought is classified as a natural phenomenon, caused mainly by a shortage of rainwater in combination with other factors of a geographical and geological nature. However, the effects of drought are felt both by humans and the environment (i.e., hunger, dust storms, altered structure of soil microbial communities) [32]. An agricultural drought is defined as a period when soil moisture is insufficient to meet the water needs of most wild plants and, most importantly, crops [33]. On the other hand, the term hydrological drought refers to the period when water flows in rivers fall below the average flow (this applies to catchment areas). In the case of a long-term meteorological drought, there is a significant reduction in groundwater levels [34]. Figure 1 graphically shows the discussed dependencies and conditions conductive for the formation of drought in the hydrological cycle, as well as the main characteristics of the individual phases of development of this phenomenon. Note that Figure 1 refers only to areas with terrestrial watercourses. Unfortunately, not all land areas are so privileged.

### 2.3. The Importance of Land Desertification

Soil plays a major role in agricultural and agro-forestry production. Therefore, any degradation of the soil quality is an extremely important environmental problem resulting in plant stress and vulnerability of classical cropping systems [33,34] Currently, we are dealing with a real ecological crisis of desertification in arid and semi-arid areas, which is causing profound local transformations of ecosystems. In some arid areas, this may even reduce the ability of some habitats to sustain life. The phenomenon of desertification is associated with long-term interactions with climatic, geochemical, biological and anthropogenic background [34,35,36], which may be studied, among various ways, by comparing maps showing the spatial and temporal distribution of land use [37] or by detecting changes through so-called pixel-based methods such as remote sensing techniques [38,39]. The surface layer of the Earth covers about 41% of dry land, and these hostile areas are inhabited by more than a third of the world’s population. This huge number of people live in areas subjected to desertification. Finally, the term desertification [40] is being defined as the process of gradual transformation of fertile land into desert as a result of increased air temperature, prolonged periods of drought, deforestation and inappropriate agricultural techniques. So, is it possible to somehow counteract this extremely unfavorable process? 

## 3. Issues That Need to Be Addressed Regarding the Recovery of Unproductive Land

### 3.1. Defining Water Retention

Drastically prolonged periods of drought result in vast areas of marginal land that are unsuitable for food production. Therefore, the first issue that should be addressed to alleviate stress and counteract economic losses is to improve the soil water retention capacity. According to the currently used classification, we distinguish between natural and artificial retention, but they remain closely related [41]. Geographically, water retention is the ability of a river basin to hold water. In general, it depends largely on the landforms, that is, the topography of the land surface, and the coverage of the land with vegetation (dense or sparse). Of course, human economic and living activities in the area under consideration are not insignificant [42]. 

Retention in the river basin is divided into five distinct groups or types. The first of these, landscape retention, depends on the topography of the area, the degree of its coverage with vegetation, the form of land development and the way it is used. The increase in the level of retained water is positively influenced by the reduction of surface runoff of rainwater. This is usually associated with infiltration, i.e., seepage of water deep into the soil profile. If the catchment is forested and, moreover, the forest has a natural character, its infiltration capacity is much greater than in a non-forested area [43]. The second type, i.e., soil retention, is the retention of water in the unsaturated zone of the soil profile. Its level depends on the type of soil, its structure and physico-chemical composition as well as on vegetation cover [44,45]. Therefore, in order to improve soil retention, we should apply the appropriate agro-technical treatments, such as fertilizing with organic compounds or other accordingly targeted treatments [32], especially those that lead to an increase in the humus content in the soil, effectively improving its structure [46]. A similar effect can be achieved by eliminating poorly permeable inter-layers or loosening soils prone to crusting. All these measures will increase soil retention [47,48]. Thirdly, groundwater and underground water retention involves the accumulation of water in an aquifer saturation zone. The amount of groundwater resources depends on the geological structure of the considered area and the degree of infiltration [49,50]. In order to increase groundwater and groundwater retention, it is necessary to limit surface runoff by applying appropriate anti-erosion measures (e.g., the creation of compact turf using grasses and some dicotyledonous herbaceous species, afforestation), and increasing soil permeability by using appropriate anti-erosion and phytomeliorative treatments [51,52,53,54]. Fourthly, surface water retention is considered the best form of water storage in natural (and artificial) reservoirs [55,56], as it significantly improves the water balance, leaving the landscape unchanged. So-called small-scale retention involves storing water in small reservoirs, both natural and artificial [57,58]. Fifth, snow and ice retention is very important [59], but does not apply to the areas in question.

### 3.2. Management of the Marginal Land

Having clarified the issues related to water retention, it would now be appropriate to briefly characterize the concept of marginal land. On this type of land, as previously mentioned, economically justified traditional crop production is not possible due to different types of environmental disturbances. The category of marginal soils includes highly erodible, desiccated sandy soils [60], acid and saline soils [61,62], sloping soils [63], compaction-prone soils [64], flood-prone soils [65], reclaimed mine soils [66,67,68], degraded lands [69], and urban soils [70,71], to mention only the most important. 

**Figure 2:**
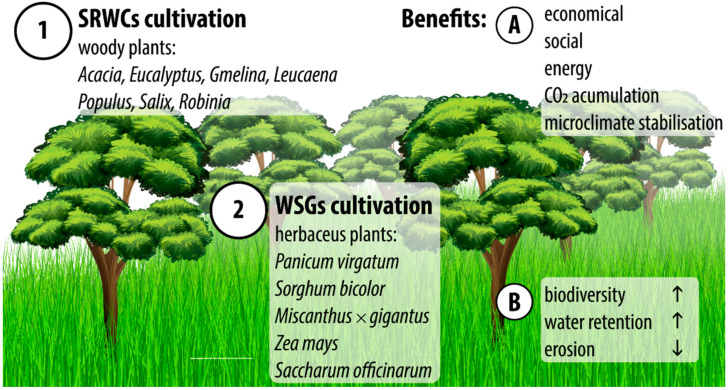
Benefits of lignocellulosic feed-stock cultivation on marginal soil. Some examples of frequently used (1) woody plants and (2) herbaceous plants, often used on arid marginal lands, which brings basic benefits from both (**A**) an utilitarian and (**B**) environmental point of view. Detailed explanation in text.

Considering physically and/or chemically destabilized soils, or those not currently cultivated due to extremely unfavorable climatic conditions, it is assumed that they show the potential for growing either warm season grasses (WSGs) or short-rotation woody crops (SRWCs) for cellulosic biomass, hemicellulose or wood (fiber or ligno-mass) production, respectively (Figure 2). This is referred to as the production of the so-called lignocellulosic feed-stocks [72,73]. In this case, to some representative examples of bioenergetic herbaceous plants useful in these cultivation systems belong *Panicum virgatum* (switchgrass), *Sorghum bicolor* (sweet sorghum), *Miscanthus* × *giganteus* (giant miscanthus), *Zea mays* (maize), and *Sacharum officinarum* (sugarcane) [74,75,76], whereas, with regard to plantation-based forestry or SRWCs, also called short-rotation forestry, short-rotation coppice or energy forestry, this type of cultivation is based upon short growth cycles (from one to about several years) with subsequent coppice regeneration. Therefore, a condition for the re-growth of trees is essential. Typically, angiosperms trees such as *Acacia*, *Eucalyptus* [77], *Gmelina* [78], and *Leucaena* [79] are used in Mediterranean, tropical and subtropical regions, while in temperate regions *Populus, Salix* [75,80,81], and *Robinia pseudoacacia* (black locust) [82] are commonly used. Both the aforementioned and other herbaceous and woody plants have the potential to improve social (labor provision) and economic (income) relations, increasing green energy production [72,73,74,75,76,77,78,79,80,81,82] while providing ecosystem services such as promoting biodiversity, carbon dioxide accumulation in plants and soil, improving water retention, reducing erosion, and stabilization of microclimatic conditions. Thus, growing this type of plant material generates multifaceted benefits for people and nature.

## 4. Some Specific Problems We Encounter in Urbanocenoses

### 4.1. Socio-Economic Aspects of Metropolitan Regions and Peri-Urban Areas

As a result of the global trend of urbanization [83,84] that has been going on for decades, there has been a significant reduction in the awareness of the human populations of numerous geographic regions (and individual countries) regarding the close relationship between our well-being and a harmoniously functioning ecosystem. Additionally, this dependence invariably remains fundamental [85]. It directly affects the well-being of residents of all kinds of administrative units, including cities and suburban regions. Moreover, most of the world’s metropolitan regions are experiencing a steady dynamic increase in population density, and urban sprawl is affecting land use patterns, the sphere of public services, and other socio-economic linkages [86,87,88]. 

In some countries of the world, especially the well-developed ones, clear socio-economic and structural transformations of suburban spaces within urban–rural regions have already been taking place since the mid-1970s. As a result, so-called peri-urban agricultural systems have been rapidly changing over increasingly large areas [89,90]. Due to this widespread phenomenon, the concept of ‘peri-urbanization’ [91] was even introduced into the scientific literature, which refers to suburban areas subjected to strong urbanization pressure. At the same time, it must be acknowledged that sometimes this pressure may have a positive impact [92], although most often it definitely projects negatively on a number of socioeconomic processes characteristic of both peri-urban neighborhoods located in the immediate vicinity of cities and the adjacent agricultural area [93,94]. Thus, peri-urban areas are experiencing fragmentation or even depletion of traditional agricultural systems [95], which modifies land values and significantly reduces community cohesion. Above all, however, unregulated urban growth results in the loss of vast areas of fertile soils with high production values [83], which in turn has a hugely negative impact on ecosystem processes and services [96,97]. In addition, as a result of uncontrolled urban sprawl in the regions in question, degenerative changes in rural landscapes are gradually taking place, especially their strong homogenization [86]. Therefore, the spatial arrangement of peripheral areas of cities and suburban villages is subject to progressive, usually unfavorable transformations [98,99]. In this perspective, it is important to have a comprehensive understanding of peri-urban agriculture and its changes over time and space. This information is necessary to undertake strategies for the sustainable management of land where the cultivation of crops is still potentially possible. Proper land use and management of such areas can only be carried out using the professional, expert knowledge of properly trained groups of specialists in this field [85,87,97,100].

### 4.2. Interdependence among Urban and Rural Communities

As a result of long-term research, it has been fully proven that urban and peri-urban societies remain largely dependent on each other, not only in the nutritional sphere but also in the ecological services provided. Consequently, detailed analyses of the dynamics of peri-urban agriculture [83,101] and its direct impact on the well-being of adjacent urban communities and even their development—through recreational opportunities for residents, the provision of beneficial visual amenities, and the regulation of soil water relations—has been effective as it has enabled the retention of water to reduce the risk of flooding in a residential area. Such studies are conducted in European countries [93,102], or other inhabited areas [103,104]. A more recent trend involves agricultural activity in urban areas.

As a result of urbanization pressures, the Earth’s vast agricultural areas have been transformed into spaces characterized by fragmented landscapes, environmental pollution and disrupted socioeconomic relations [93,103]. The Food and Agriculture Organization of the United Nations has published a report reporting that, according to the latest projections, food can be provided for up to eight billion people on Earth, assuming that the ever-increasing process of soil degradation is stopped in the short term, and degraded land is brought to a state that allows it to be developed to allow for agricultural activities. World demographic projections assume that the population will exceed this number as early as 2025 (www.unpopulation.org, accessed on 18 December 2022), and in 2050 it is estimated that the population could exceed ten billion [105]. 

### 4.3. Urban Agriculture

In cities, Nature-Based Solutions (NBS), defined as animate nature-inspired projects to address various environmental, economic and social challenges, are a favorable solution to the problem [106,107,108,109]. For this reason, the activity of urban and peri-urban residents administratively and functionally linked to cities, referred to as “urban agriculture” [110], sometimes also called urban horticulture [108], agriurbanism, or metropolitan agricultural production, has been promoted in recent decades [93,103,111,112]. Such activity can also be understood as a professional or social activity conducted within the city limits, during which various types of agricultural products are produced, usually for the local urban market [104,113]. In broad terms, urban agriculture refers to the practice of growing crops in urban spaces, so the appropriate design of urban agriculture requires a certain level of expertise in agricultural and horticultural sciences [112,114].

Thus, when considering the importance of environmental manaagement measures to restore multifunctional urban spaces that ensure the well-being of all residents, it is important to consider the case of urban gardening. Of course, on the other hand, properly designed green architecture is gaining popularity nowadays and more and more people pay attention to the aesthetic aspect of cities [115]. Wuzhong [116] promoted the so-called 3A (Agriculture, Architecture, Art) concept, which is a combination of knowledge and practice (experience) of horticulture, architecture and art in the process of shaping the modern face of cities. 

## 5. Is Regenerative Agriculture Really the Solution to the Problem of Suburban Areas and Marginal Lands?

### 5.1. Brownfield Reclamation to Create Social Connections

It should be noted that so-called community gardens are seen as an effective way to generate social integration on suburban areas [117]. Published information on the subject emphasized the therapeutic role of creating social connections and the benefits of building a community that is aware of the need to reduce anthropopressure [118] and actively engage in environmentally friendly activities [119,120]. In addition, agriculture or horticulture in brownfield sites is a remedy for social fragmentation, while providing an effective way of working and effectively earning money [121]. People involved in agricultural production generate beneficial changes for their community. The openness of projects of this type, with the use of self-help for the individuals involved in the project, effectively leads to the economic restructuring of human populations. This occurs mainly through greater access to the public sphere for those people formerly alienated from the community [93]. 

Positive changes take place in the aesthetics of the areas [122], while the sense of safety and health of the local residents grows. Simultaneously, plants reduce air pollution [123]. In addition, the benefits of gardening have a positive effect on people’s physical and mental health. A relatively new field of knowledge is horticultural therapy [124] which has a beneficial effect on human health and even facilitates the adaptation of patients to a period of physical disability.

Since it is also possible to consider agricultural activity in terms of a social process in which individuals participate, so all local initiatives to grow various types of plants, not only with the intention of producing food, are of considerable importance. Therefore, nowadays, this branch of activity is once again in a period of great boom. Additionally, for this reason, such activity should be carried out on marginal land. Then, you can interact positively for the community and nature. At this point, the term regenerative agriculture should be used, which is the best approach to reclaiming marginal land, regenerating the soil and preserving compact soil cover. At the same time, biodiversity increases, the water cycle improves, and ecosystem services are enhanced [125]. Consequently, resilience to climate change is significantly increasing. Thus, there is no alternative; such projects must be undertaken.

### 5.2. Plant Choice for Saline Marginal Land

Since there have been water deficits in recent decades (and this will not change in the future), the emergence of crops grown by sowing, such as cereals, has been delayed and in the later stages of development drought increases intra-species competition in the canopy, resulting in low crop productivity. Therefore, there is growing interest around the world in sustainable agriculture which aims to increase plant biodiversity in field. The popularity of mixed crops typical of regenerative agriculture is due, among other things, to their higher yield potential compared to classic agricultural crops, establishing monocultures. In Table 1, we collected purposely selected species, as there are of course many more that can be applied to marginal lands affected by various stress factors, namely water deficit stress and salinity stress. This exemplary selection is meant to be used in different climatic regions of the world. Additionally, salinity can also result from the use of desalinated seawater or pre-treated water from human habitation activities. It must be admitted that it is quite difficult in a small selection, as can be seen from the given examples of herbaceous and woody plants collected in the table, to present all possible permutations of species suitable for all categories of marginal soils. In addition, soilless areas, such as desert sands, could also be considered. However, this would require an individual approach to a specific site, with its geology, substrate chemistry and climatic conditions that exist there. Additionally, this would perhaps require a separate publication that would deal only with these examples. 

Furthermore, the yield of herbaceous and woody mixtures depends on a number of factors, the most important of which are the choice of species components, the proportion of plants that enter into biotic relations with microorganisms in the mixture, the fertility of the substrate and weather conditions. This is a tremendously wide range of factors. We plan to address scientific issues of this kind in a project we intend to implement soon in specific marginal areas in the near future.

## 6. Conclusions

The review performed allowed the authors to conclude that various forms of green infrastructure play an important role in humidifying, purifying and cooling the microclimate and, at the same time, play an important role in the aesthetics of the landscape in previously derelict land. The authors suggested that regenerative agriculture could provide the habitat necessary to increase diversity. The current state of knowledge and the degree to which theory is organized and integrated in the service of practice does not allow for the formulation of clear and ready-made guidelines for practical global action. Each reclamation site should be considered separately in terms of geophysical and climatic conditions and the range of plant species that could be used under these conditions. A different range of species should be applied in urban greenery, and another, much broader, in both open suburban areas and on marginal lands of various types. Former agricultural land offers a wider range of approaches, and this is where regenerative agriculture can be most successful.

## Figures and Tables

**Table 1:** Examples of herbaceous and woody plants suitable for growth in arid areas on saline marginal land under agricultural conditions.

Species	Family	Growth Habit	Notes	References
*Armeria maritima* (Mill.) Willd.	Plumbaginaceae	HP ^1^	Very well adapted to arid, saline areas	[126]
*Limonium vulgare* Mill.	Plumbaginaceae	HP	Very well adapted to arid, saline area; Edible plant	[126,127]
*Limonium tetragonum* (Thunb.) Bullock	Plumbaginaceae	HP	Very well adapted to arid, saline areas; Edible plant	[127]
*Atriplex halimus* L.	Chenopodiaceae	HP	Very well adapted to arid, calcareous sodic soil	[128]
*Atriplex lentiformis* (Torr.) Wats	Chenopodiaceae	HP	Very well adapted to arid, saline areas; Edible	[129]
*Atriplex nummularia* Lindl.	Chenopodiaceae	HP	Very well adapted to semi-arid, saline area; Fodder for animals	[130,131]
*Mimosa caesalpinifolia* Beneth.	Leguminosae, Mimosoidae	WP ^2^	Well adapted to semi-arid area; Fodder for animals; Used as briquettes	[131,132]
*Robinia pseudoacacia*	Leguminosae	WP	Well adapted to semi-arid saline area;	[133]
*Tamarix ramosisima* Lebb.	Tamarixaceae	WP	Very well adapted to arid saline area	[134]
*Tamarix hispida* Willd.	Tamarixaceae	WP	Very well adapted to arid saline area	[135]
*Elaeagnus angustifolia* L.	Ealeagnaceae	WP	Very well adapted to arid area and saline soils	[136]
*Populus deltoides’ Populus nigra*	Salicaceae	WP	Hybrid clone selected as tolerant to arid area and saline soils	[136]
*Salix psamophila*	Salicaceae	WP	Very well adapted to arid area and highly tolerant to salty soil	[137]

^1^ herbaceous plant, ^2^ woody plant.

## Data Availability

Not applicable.
